# Supplementary material for: Glaucoma through Animal’s Eyes: Insights from the Evolution of Intraocular Pressure in Mammals and Birds
Source: Animals (Basel). 2022 Aug 10;12(16):2027. doi: 10.3390/ani12162027 (PMC9404445; doi:10.3390/ani12162027)
Supplement: Supplementary file 1 [file animals-12-02027-s001.zip › Supplementary document 1.pdf]

1. Adelman, S., Shinsako, D., Kiland, J. A., Yaccarino, V., Ellinwood, N. M., Ben-Shlomo, G., & McLellan, G. J. (2018). The post-natal development of intraocular pressure in normal domestic cats (Felis catus) and in feline congenital glaucoma. *Experimental eye research, 166*, 70-73. https://doi.org/10.1016/j.exer.2017.10.016
2. Afrashi, F., Karatepe Hashas, A. S., Shahbazov, C., Arici, M., Yikilmaz, M. S., Deveci, R., . . . Sahar, U. (2015). Reliability of intravitreal nepafenac in rabbits. *Journal of Ocular Pharmacology and Therapeutics, 31*(1), 43-50.
3. Agarwal, R., & Agarwal, P. (2017). Rodent models of glaucoma and their applicability for drug discovery. *Expert opinion on drug discovery*, *12*(3), 261-270.
4. Akaishi, T., Shimazaki, A., Tonouchi, A., Ueda, K., Miyawaki, N., & Kawazu, K. (2015). Benefits of tafluprost and timolol fixed-dose combination for the treatment of glaucoma are confirmed by studies on experimental animal models. *Journal of Ocular Pharmacology and Therapeutics, 31*(9), 518-524.
5. Al-Sobayil, F. A., Ahmed, A. F., Al-Wabel, N. A., Al-Thonayian, A. A., Al-Rogibah, F. A., Al-Fuaim, A. H., . . . Al-Muzaini, A. M. (2009). The use of xylazine, ketamine, and isoflurane for induction and maintenance of anesthesia in ostriches (Struthio camelus). *Journal of Avian Medicine and Surgery*, *23*(2), 101-107.
6. Alkozi, H., Sánchez-Naves, J., de Lara, M. J. P., Carracedo, G., Fonseca, B., Martinez-Aguila, A., & Pintor, J. (2017). Elevated intraocular pressure increases melatonin levels in the aqueous humour. *Acta Ophthalmologica*, *95*(3), e185-e189.
7. Alsarraf, O., Fan, J., Dahrouj, M., Chou, C. J., Yates, P. W., & Crosson, C. E. (2014). Acetylation preserves retinal ganglion cell structure and function in a chronic model of ocular hypertension. *Investigative Ophthalmology & Visual Science, 55*(11), 7486-7493.
8. Amaral, A. V. C. d., Silva, G. A. d., Costa, A. P. A., Coelho, C. M. M., Renzo, R., & Laus, J. L. (2014). Sildenafil citrate on retrobulbar and retinal circulation of rabbits. *Ciência Rural*, *44*, 1431-1436.
9. Amorim, T. M., Dower, N. M. B., Stocco, M. B., Pizoni, L. H., & Ribeiro, A. P. (2019). Effects of intracameral injection of epinephrine and 2% lidocaine on pupil diameter, intraocular pressure, and cardiovascular parameters in healthy cats. *Veterinary ophthalmology*, *22*(3), 276-283.
10. Ancrenaz, M., Ostrowski, S., Anagariyah, S., & Delhomme, A. (1996). Long-duration anesthesia in arabian oryx (Oryx leucoryx) using a medetomidine: etorphine combination. *Journal of Zoo and Wildlife Medicine*, 209-216.
11. Andrade, M. C. C. d., Hünning, P. S., Pereira, F. Q., Dutra, K. P., & Pigatto, J. A. T. (2016). Lip twitch restraint on rebound tonometry in horses. *Ciência Rural*, *46*, 1486-1490.
12. Ariza-Gracia, M., Ortillés, Á., Cristóbal, J., Matas, J. F. R., & Calvo, B. (2017). A numerical-experimental protocol to characterize corneal tissue with an application to predict astigmatic keratotomy surgery. *Journal of the Mechanical Behavior of Biomedical Materials*, *74*, 304-314.
13. Awad, R., Nocera, I., Briganti, A., Khaled, A., Sabry, E.-K., Michihito, T., & Bonelli, F. (2019). Dose-dependent effect of romifidine on intraocular pressure in clinically healthy buffalo (Bubalus Bubalis).
14. Bai, Y., Zhu, Y., Chen, Q., Xu, J., Marinko, V. S., Uri, H. S., & Zhuo, Y. (2014). Validation of glaucoma-like features in the rat episcleral vein cauterization model. *Chinese medical journal*, *127*(02), 359-364.

15. Bailey, J. E. Arterial Blood Pressure Monitoring of Select Pinnipeds: Multi-Case Presentation IAAAM 2013.
16. Balthazar da Silveira, C. P., Lima, T. B., Crivelaro, R. M., de Lacerda, L. C. C., Pádua, I. R. M., Renzo, R., . . . Laus, J. L. (2018). Ophthalmic parameters in adult lowland paca (Cuniculus paca) raised in captivity. *Veterinary ophthalmology*, *21*(1), 42-47.
17. Barsotti, G., Asti, M., Giani, E., Ceccherelli, R., & Briganti, A. (2019). Effect of topical ophthalmic instillation of rocuronium bromide on the intraocular pressure of kestrels (Falco tinnunculus) and little owls (Athene noctuae). *Journal of the American Veterinary Medical Association*, *255*(12), 1359-1364.
18. Bebarta, V. S., Tanen, D. A., Boudreau, S., Castaneda, M., Zarzabal, L. A., Vargas, T., & Boss, G. R. (2014). Intravenous cobinamide versus hydroxocobalamin for acute treatment of severe cyanide poisoning in a swine (Sus scrofa) model. *Annals of emergency medicine*, *64*(6), 612-619.
19. Becker, S., Reinehr, S., Dick, H. B., & Joachim, S. C. (2015). Complement activation after induction of ocular hypertension in an animal model. *Der Ophthalmologe: Zeitschrift der Deutschen Ophthalmologischen Gesellschaft*, *112*(1), 41-48.
20. Bello, S. A., & Passaglia, C. L. (2017). A wireless pressure sensor for continuous monitoring of intraocular pressure in conscious animals. *Annals of biomedical engineering*, *45*(11), 2592-2604.
21. Bettschart-Wolfensberger, R., Semder, A., Alibhai, H., Demuth, D., Shojaee Aliabadi, F., & Clarke, K. W. (2000). Cardiopulmonary side-effects and pharmacokinetics of an emulsion of propofol (Disoprivan®) in comparison to propofol solved in polysorbate 80 in goats. *Journal of Veterinary Medicine Series A, 47*(6), 341-350.
22. Bliss, C. D., Aquino, S., & Woodhouse, S. (2015). Ocular findings and reference values for selected ophthalmic diagnostic tests in the macaroni penguin (E udyptes chrysolophus) and southern rockhopper penguin (E udyptes chrysocome). *Veterinary Ophthalmology*, *18*, 86-93.
23. Boucher, C. J., Venter, I. J., Tordiffe, A. S. W., & Kirberger, R. M. (2019). INTRAOCULAR PRESSURE, TEAR PRODUCTION, AND OCULAR BIOMETRY IN HEALTHY ADULT CHEETAHS (ACINONYX JUBATUS). *Journal of Zoo and Wildlife Medicine*, *50*(3), 634-643. https://doi.org/10.1638/2018-0156
24. Brown, S., Atkins, C., Bagley, R., Carr, A., Cowgill, L., Davidson, M., . . . Labato, M. (2007). Guidelines for the identification, evaluation, and management of systemic hypertension in dogs and cats. *Journal of veterinary internal medicine*, *21*(3), 542-558.
25. Cairó, M., Peña, M. T., Rios, J., Melero, A., Martorell, J., & Leiva, M. (2018). Assessment of intraocular pressure by applanation and rebound tonometry in guinea pigs of different ages. *Journal of Exotic Pet Medicine*, *27*(1), 25-31.
26. Carvalho, C. M., Rodarte-Almeida, A. C. V., Beanes, A. S., Machado, M. T. S., & Galera, P. D. (2020). Ophthalmic contributions to assessing eyes of two neotropical canids: Cerdocyon thous and Chrysocyon brachyurus. *Veterinary ophthalmology*, *23*(3), 460-471.
27. Chacaltana, F. D. Y. C., Pigatto, J. A. T., & Denardin, I. T. (2016). Assessment of intraocular pressure in chinchillas of different age groups using rebound tonometry. *Ciência Rural*, *46*, 1466-1471.

28. Chen, H.-F., Chen, M.-C., Lai, C.-C., Yeung, L., Wang, N.-K., Chen, H. S.-L., . . . Chuang, L.-H. (2014). Neovascular glaucoma after central retinal vein occlusion in pre-existing glaucoma. *BMC ophthalmology*, *14*(1), 1-6.
29. Chen, S., & Zhang, X. (2015). The rodent model of glaucoma and its implications. *The Asia-Pacific Journal of Ophthalmology*, *4*(4), 236-241.
30. Colitz, C. M., Mejia-Fava, J., Yamagata, M., Smolensky, P., Renner, M. S., Haulena, M., . . . Orona, J. F. (2012). Preliminary intraocular pressure measurements from 4 cetacean species.
31. Comez, A. T., Cakir, D. U., Tutunculer, F. K., Gencer, B., & Tufan, H. A. (2014). Relationship between raised intraocular pressure and ischemia-modified albumin in serum and humor aqueous: a pilot study in rabbits. *International Journal of Ophthalmology*, *7*(3), 421.
32. Costa, D., Leiva, M., Coyo, N., Laguna, F., Ríos, J., & Peña Gimenez, M. T. (2016). Effect of topical 1% cyclopentolate hydrochloride on tear production, pupil size, and intraocular pressure in healthy Beagles. *Veterinary ophthalmology*, *19*(6), 449-453.
33. Dalvin, L. A., & Fautsch, M. P. (2015). Analysis of circadian rhythm gene expression with reference to diurnal pattern of intraocular pressure in mice. *Investigative ophthalmology & visual science*, *56*(4), 2657-2663.
34. de Crom, R. M. P. C., Webers, C. A. B., van Kooten-Noordzij, M. A. W., Michiels, A. C., Schouten, J. S. A. G., Berendschot, T. T. J. M., & Beckers, H. J. M. (2017). Intraocular pressure fluctuations and 24-hour continuous monitoring for glaucoma risk in wind instrument players. *Journal of glaucoma*, *26*(10), 923-928.
35. Di, Y., Luo, X.-M., Qiao, T., & Lu, N. (2017). Intraocular pressure with rebound tonometry and effects of topical intraocular pressure reducing medications in guinea pigs. *International Journal of Ophthalmology*, *10*(2), 186.
36. Diehl, K., & Bowden, A. C. (2020). Effect of auriculopalpebral nerve block on equine intraocular pressure measured by rebound tonometry (TonoVet®). *Veterinary ophthalmology*, *23*(2), 368-373.
37. DiGeronimo, P. M., Pisano, S. R. R., Di Girolamo, N., Spielvogel, C. F., Pirie, G. J., & Carter, R. (2018). Selected ophthalmic parameters and potential risk for light-induced cataracts in two colonies of captive indian flying foxes (pteropus giganteus). *Journal of zoo and wildlife medicine*, *49*(1), 129-133.
38. Dominguez, R. (1927). The systolic blood pressure of the normal rabbit measured by a slightly modified van Leersum method. *The Journal of Experimental Medicine*, *46*(3), 443-461.
39. Doyle, J. T., Patterson Jr, J. L., Warren, J. V., & Detweiler, D. K. (1960). Observations on the circulation of domestic cattle. *Circulation Research*, *8*(1), 4-15.
40. Erol, H., & Arican, M. (2019). The investigation of xylazine, detomidine, isoflurane and sevoflurane anaesthetic combinations on clinical, laboratory and cardiovascular parameters and on intraocular pressure in horses. *Journal of the Hellenic Veterinary Medical Society*, *70*(1).
41. Gatson, B. J., Paranjape, V., Wellehan, J. F. X., & Bailey, K. (2019). A description of arterial blood pressure measurement in two species of flying foxes (Pteropus vampyrus and Pteropus hypomelanus). *Journal of Zoo and Wildlife Medicine*, *50*(3), 665-671.

42. Gonzalez-Alonso-Alegre, E. M., Martinez-Nevado, E., Caro-Vadillo, A., & Rodriguez-Alvaro, A. (2015). Central corneal thickness and intraocular pressure in captive black-footed penguins (S pheniscus dermersus). *Veterinary ophthalmology*, *18*, 94-97.
43. Hasiuk, M. M. M., Forde, N., Cooke, A., Ramey, K., & Pang, D. S. J. (2014). A comparison of alfaxalone and propofol on intraocular pressure in healthy dogs. *Veterinary ophthalmology*, *17*(6), 411-416.
44. Heard, D. J., & Beusse, D. O. (1993). Combination detomidine, ketamine, and isoflurane anesthesia in California sea lions (Zalophus californianus). *Journal of Zoo and Wildlife Medicine*, 168-170.
45. Heard, D. J., Olsen, J. H., & Stover, J. (1992). Cardiopulmonary changes associated with chemical immobilization and recumbency in a white rhinoceros (Ceratotherium simum). *Journal of Zoo and Wildlife Medicine*, 197-200.
46. Hibbs, C. D., Barrett, P. M., & Dees, D. D. (2019). Intraocular pressure reference intervals in eyes of clinically normal miniature donkeys (Equus africanus asinus). *Veterinary ophthalmology*, *22*(1), 24-30.
47. Honsho, C. S., Jorge, A. T., Oliveira, L. T., Paulino-Junior, D., Mattos-Junior, E., Nishimura, L. T., & Dias, W. O. (2016). Intraocular pressure and Schirmer tear test values in maned wolf (Chrysocyon brachyurus). *Pesquisa Veterinária Brasileira*, *36*, 919-923.
48. Janssen, D. L., Swan, G. E., Raath, J. P., McJames, S. W., Allen, J. L., de Vos, V., . . . Stanley, T. H. (1993). Immobilization and Physiologic Effects of the Narcotic A-3080 in Impala (Aepyceros melampus). *Journal of Zoo and Wildlife Medicine*, *24*(1), 11-18.
49. Kadden, R. M., Schoenfeld, W. N., McCullough, M. R., Steele, W. A., & Tremont, P. J. (1980). Classical conditioning of heart rate and blood pressure in Macaca mulatta. *Journal of the Autonomic Nervous System*, *2*(2), 131-142.
50. Kampmeier, T., Arnemann, P., Heßler, M., Rehberg, S., Morelli, A., Westphal, M., . . . Ertmer, C. (2017). Provision of physiological data and reference values in awake and anaesthetized female sheep aged 6–12 months. *Veterinary anaesthesia and analgesia*, *44*(3), 518-528.
51. Kanda, T., Iguchi, A., Yoshioka, C., Nomura, H., Higashi, K., Kaya, M., . . . Furukawa, T. (2015). Effects of medetomidine and xylazine on intraocular pressure and pupil size in healthy Beagle dogs. *Veterinary Anaesthesia and Analgesia*, *42*(6), 623-628.
52. Kim, J., Kim, D., Kim, E.-j., Lee, H.-b., Kim, N.-S., & Kim, M.-S. (2017). Ophthalmic examination in common kestrels (Falco tinnuculus) from South Korea. *Journal of Zoo and Wildlife Medicine*, *48*(3), 683-687.
53. Kinney, M. E., Ericsson, A. C., Franklin, C. L., Whiting, R. E. H., & Pearce, J. W. (2017). Ocular findings and select ophthalmic diagnostic tests in captive american white pelicans (Pelecanus erythrorhynchos). *Journal of zoo and wildlife medicine: official publication of the American Association of Zoo Veterinarians*, *48*(3), 675.
54. Kovalcuka, L., Boiko, D., & Williams, D. L. (2018). Tear production and intraocular pressure values in clinically normal eyes of whooper swans (Cygnus cygnus). *Open Veterinary Journal*, *8*(3), 335-339.
55. Kshirsagar, A. V., Carpenter, M., Bang, H., Wyatt, S. B., & Colindres, R. E. (2006). Blood pressure usually considered normal is associated with an elevated risk of cardiovascular disease. *The American journal of medicine*, *119*(2), 133-141.

56. Kulualp, K., Kiliç, S., Cakir, S., & Orhan, C. (2018). Evaluation of intraocular pressure (IOP) regarding circadian rhythm, age, sex and eye side in Awassi sheep. *Journal of the Hellenic Veterinary Medical Society*, *69*(2), 959-964.
57. Kurt, B., Aksoy, Ö., & Çağatay, H. H. (2018). The effect of body position on intraocular pressure in calves. *Journal of the South African Veterinary Association*, *89*(1), 1-3.
58. Kuwahara, M., Yagi, Y., Ji, B., Si, S., Tsubone, H., Sugano, S., & Kobayashi, H. (1996). Non-invasive measurement of systemic arterial pressure in guinea pigs by an automatic oscillometric device. *Blood Pressure Monitoring*, *1*(5), 433-437.
59. Kvapil, P., Pirš, T., Slavec, B., Luštrik, R., Zemljič, T., Bártová, E., . . . Kastelic, M. (2018). Tear production, intraocular pressure and conjunctival bacterial flora in selected captive wild ruminants. *Veterinary ophthalmology*, *21*(1), 52-57.
60. Lapid, R., King, R., Bdolah-Abram, T., & Shilo-Benjamini, Y. (2017). A retrospective comparison of chemical immobilization with thiafentanil, thiafentanil-azaperone, or etorphine-acepromazine in captive Persian fallow deer (Dama dama mesopotamica). *Journal of Zoo and Wildlife Medicine*, *48*(3), 627-635.
61. Lapid, R., & Shilo-Benjamini, Y. (2015). Immobilization of captive Nubian ibex (Capra nubiana) with butorphanol-midazolam-medetomidine or butorphanol-azaperone-medetomidine and atipamezole reversal. *Journal of Zoo and Wildlife Medicine*, *46*(2), 291-297.
62. Luo, Y., Owens, D., Mulder, G., McVey, A., & Fisher, T. (2008). Blood pressure characterization of hypertensive and control rats for cardiovascular studies. *AHA, Atlanta: Charles River*.
63. Mama, K. R., Phillips Jr, L. G., & Pascoe, P. J. (1996). Use of propofol for induction and maintenance of anesthesia in a barn owl (Tyto alba) undergoing tracheal resection. *Journal of Zoo and Wildlife Medicine*, 397-401.
64. Marzok, M. A., El-khodery, S. A., & Oheida, A. H. (2014). Effect of intravenous administration of romifidine on intraocular pressure in clinically normal horses. *Veterinary ophthalmology*, *17*, 149-153.
65. Meekins, J. M., Eshar, D., & Rankin, A. J. (2015). Tear production, intraocular pressure, and conjunctival bacterial flora in a group of captive black-tailed prairie dogs (C ynomys ludovicianus). *Veterinary Ophthalmology*, *18*, 132-136.
66. Miller, R. E., & Buss, P. E. (2003). Rhinoceridae (rhinoceroses). *Zoo and Wild Animal Medicine. St. Louis, MO: Saunders,* 558-569.
67. Monção-Silva, R., Ofri, R., Raposo, A. C., Araújo, N., Torezani, J., Muramoto, C., & Oriá, A. (2016). Ophthalmic diagnostic tests in parrots (Amazona amazonica) and (Amazona aestiva). *Journal of Exotic Pet Medicine*, *25*(3), 186-193.
68. Oriá, A. P., Gomes Junior, D. C., Oliveira, A. V. D., Curvelo, V. P., Estrela-Lima, A., Pinna, M. H., . . . Ofri, R. (2015). Selected ophthalmic diagnostic tests, bony orbit anatomy, and ocular histology in sambar deer (R usa unicolor). *Veterinary Ophthalmology*, *18*, 125-131.
69. Ostrin, L. A., & Wildsoet, C. F. (2016). Optic nerve head and intraocular pressure in the guinea pig eye. *Experimental eye research*, *146*, 7-16.
70. Pacheco, R. E., Bauer, B. S., & Sadar, M. J. (2018). Measurement of tear production and intraocular pressure in conscious captive European fallow deer (DAMA dama). *Veterinary Medicine and Science*, *4*(3), 227-236.

71. Peche, N., & Eule, J. C. (2018). Intraocular pressure measurements in cattle, sheep, and goats with 2 different types of tonometers. *Canadian Journal of Veterinary Research, 82*(3), 208-215.
72. Pietro, S. d., Passantino, A., Crinò, C., Rizzo, M., Giannetto, C., & Giudice, E. (2016). Comparison of tear production and intraocular pressure in domestic and wild ruminants. *Large Animal Review, 22*(1), 39-42.
73. Rajaei, S. M., Mood, M. A., & Hashemi, S. S. G. (2016). Measurement of tear production and intraocular pressure in healthy captive helmeted guinea fowl (Numida meleagris). *Journal of Avian Medicine and Surgery*, 324-328.
74. Rajaei, S. M., Mood, M. A., Sadjadi, R., & Williams, D. L. (2016). Results of selected ophthalmic diagnostic tests for clinically normal Syrian hamsters (Mesocricetus auratus). *American Journal of Veterinary Research, 77*(1), 72-76.
75. Rey, B., Costello, M.-A., Fuller, A., Haw, A., Hetem, R. S., Mitchell, D., & Meyer, L. C. R. (2014). Chemical immobilization and anesthesia of free-living aardvarks (Orycteropus afer) with ketamine-medetomidine-midazolam and isoflurane. *Journal of Wildlife Diseases, 50*(4), 864-872.
76. Romagnoli, A. (1956). Indirect Blood Pressure Measurement in Sheep and Goats Employing the Electronic Plethysmograph:: Validation against the Capacitance Manometer. *British Veterinary Journal, 112*(6), 247-252.
77. Schellenberg, S., Glaus, T. M., & Reusch, C. E. (2007). Effect of long-term adaptation on indirect measurements of systolic blood pressure in conscious untrained beagles. *Veterinary record, 161*(12), 418-421.
78. Semjonov, A., Andrianov, V., Raath, J. P., Orro, T., Venter, D., Laubscher, L., & Pfitzer, S. (2017). Evaluation of BAM (butorphanol–azaperone–medetomidine) in captive African lion (Panthera leo) immobilization. *Veterinary anaesthesia and analgesia, 44*(4), 883-889.
79. Sheldon, J. D. Measurement of Intraocular Pressures Using Rebound Tonometry in South American Fur Seals (Arctocephalus australis) and South American Sea Lions (Otaria flavescens) from Punta San Juan, Peru IAAAM 2017.
80. Siegal-Willott, J., Citino, S. B., Wade, S., Elder, L., Hayek, L.-A. C., & Lance, W. R. (2009). Butorphanol, azaperone, and medetomidine anesthesia in free-ranging white-tailed deer (Odocoileus virginianus) using radiotransmitter darts. *Journal of wildlife Diseases, 45*(2), 468-480.
81. Simpson, R. U., Hershey, S. H., & Nibbelink, K. A. (2007). Characterization of heart size and blood pressure in the vitamin D receptor knockout mouse. *The Journal of steroid biochemistry and molecular biology, 103*(3-5), 521-524.
82. Thomas, C. L., Artwohl, J. E., Suzuki, H., Gao, X.-p., White, E., Saroli, A., . . . Rubinstein, I. (1997). Initial characterization of hamsters with spontaneous hypertension. *Hypertension, 30*(2), 301-304.
83. Varela-Lopez, O., Gomez-Martinez, M. I., Rodriguez, A. A., & González-Cantalapiedra, A. (2021). Immobilization of mouflon (Ovis orientalis musimon) using medetomidine–ketamine–morphine or dexmedetomidine–ketamine–morphine. *Journal of Zoo and Wildlife Medicine, 52*(3), 1018-1023.
84. Villar, T., Pascoli, A. L., Klein, A., Chacaltana, F. C., Capistrano, E., Shipley, C. F., & Martins, B. C. (2020). Tear production, intraocular pressure, and central corneal

thickness in white-tailed deer (Odocoileus virginianus). *Veterinary ophthalmology*, *23*(1), 123-128.

85. Zouache, M. A., Eames, I., & Samsudin, A. (2016). Allometry and Scaling of the Intraocular Pressure and Aqueous Humour Flow Rate in Vertebrate Eyes. *PloS one*, *11*(3), e0151490-e0151490. https://doi.org/10.1371/journal.pone.0151490
86. Andrew, S. E., Ramsey, D. T., Hauptman, J. G., & Brooks, D. E. (2001). Density of corneal endothelial cells and corneal thickness in eyes of euthanatized horses. American Journal of Veterinary Research, 62(4), 479-482.
87. Gilger, B. C., Wright, J. C., Whitley, R. D., & McLaughlin, S. A. (1993). Corneal thickness measured by ultrasonic pachymetry in cats. American journal of veterinary research, 54(2), 228-230.
88. Liang, D., Alvarado, T. P., Oral, D., Vargas, J. M., Denena, M. M., & McCulley, J. P. (2005). Ophthalmic examination of the captive western lowland gorilla (Gorilla gorilla gorilla). Journal of zoo and wildlife medicine, 36(3), 430-433.
89. Doughty, M. J., & Zaman, M. L. (2000). Human corneal thickness and its impact on intraocular pressure measures: a review and meta-analysis approach. Survey of ophthalmology, 44(5), 367-408.
90. Montiani-Ferreira, F., Truppel, J., Tramontin, M. H., D'Octaviano Vilani, R. G., & Lange, R. R. (2008). The capybara eye: clincial tests, anatomic and biometric features. Veterinary Ophthalmology, 11(6), 386-394.
91. Andrew, S. E., Willis, A. M., & Anderson, D. E. (2002). Density of corneal endothelial cells, corneal thickness, and corneal diameters in normal eyes of llamas and alpacas. American Journal of Veterinary Research, 63(3), 326-329.
92. Madigan, M. C., Gillard-Crewther, S., Kiely, P. M., Crewther, D. P., Brennan, N. A., Efron, N., & Holden, B. A. (1987). Corneal thickness changes following sleep and overnight contact lens wear in the primate (Macaca fascicularis). Current eye research, 6(6), 809-815.
93. Ollivier, F. J., Brooks, D. E., Komaromy, A. M., Kallberg, M. E., Andrew, S. E., Sapp, H. L., ... & Dawson, W. W. (2003). Corneal thickness and endothelial cell density measured by non-contact specular microscopy and pachymetry in Rhesus macaques (Macaca mulatta) with laser-induced ocular hypertension. Experimental Eye Research, 76(6), 671-677.
94. King, R., Struebing, F. L., Li, Y., Wang, J., Koch, A. A., Cooke Bailey, J. N., ... & Geisert, E. E. (2018). Genomic locus modulating corneal thickness in the mouse identifies POU6F2 as a potential risk of developing glaucoma. PLoS genetics, 14(1), e1007145.
95. Montiani-Ferreira, F., Mattos, B. C., & Russ, H. H. A. (2006). Reference values for selected ophthalmic diagnostic tests of the ferret (Mustela putorius furo). Veterinary Ophthalmology, 9(4), 209-213.
96. Paszta, W., Klećkowska-Nawrot, J. E., & Goździewska-Harłajczuk, K. (2022). Morphological evaluation of the orbit, eye tunics, eyelids, and orbital glands in young and adult aardvarks Orycteropus afer, Pallas, 1766 (Tubulidentata: Orycteropodidae): Similarities and differences with representatives of the Afrotheria clade. The Anatomical Record.

97. Chan, T., Payor, S., & Holden, B. A. (1983). Corneal thickness profiles in rabbits using an ultrasonic pachometer. Investigative ophthalmology & visual science, 24(10), 1408-1410.
98. Coyo, N., Peña, M. T., Costa, D., Ríos, J., Lacerda, R., & Leiva, M. (2016). Effects of age and breed on corneal thickness, density, and morphology of corneal endothelial cells in enucleated sheep eyes. Veterinary Ophthalmology, 19(5), 367-372.
99. C. Myers, R., Ballantyne, B., Christopher, S. M., Chun, J.S. (1998). Comparative evaluation of several methods and conditions for the in vivo measurement of corneal thickness in rabbits and rats. Toxicology Methods, 8(3), 219-231.
100. Faber, C., Scherfig, E., Prause, J. U., & Sørensen, K. E. (2008). Corneal thickness in pigs measured by ultrasound pachymetry in vivo. Scandinavian Journal of Laboratory Animal Sciences, 35(1), 39-43.
101. Lau, R. K., Moresco, A., Woods, S. J., Reilly, C. M., Hawkins, M. G., Murphy, C. J., ... & Freeman, K. S. (2017). Presumptive keratoglobus in a great horned owl (Bubo virginianus). Veterinary ophthalmology, 20(6), 560-567.
102. Jones, M. P., & Ward, D. A. (2012). Fluorophotometric determination of aqueous humor flow rates in red-tailed hawks (Buteo jamaicensis). American journal of veterinary research, 73(4), 551-555.
103. Werther, K., Candioto, C. G., & Korbel, R. (2017). Ocular histomorphometry of free-living common kestrels (Falco tinnunculus). Journal of avian medicine and surgery, 31(4), 319-326.
104. Gonzalez-Alonso-Alegre, E. M., Martinez-Nevado, E., Caro-Vadillo, A., & Rodriguez-Alvaro, A. (2015). Central corneal thickness and intraocular pressure in captive black-footed penguins (S pheniscus dermersus). Veterinary ophthalmology, 18, 94-97.
105. Pérez Orrico, M. L., & Sabater González, M. (2022). Ophthalmology of Palaeognathae: Ostriches, Rheas, Emu, Cassowaries, Tinamous, and Kiwis. Wild and Exotic Animal Ophthalmology, 627-648.
106. Boucher, C. J. (2017). *Prevalence of ocular pathology in adult captive cheetahs (Acinonyx jubatus)* (Doctoral dissertation, University of Pretoria).
107. Kurt, B. K. (2021). Central corneal thickness increases with age in cattle. Large Animal Review, 27(4), 195-198.
108. Kassab, A. (2012). Ultrasonographic and macroscopic anatomy of the enucleated eyes of the buffalo (Bos bubalis) and the one-humped camel (Camelus dromedarius) of different ages. Anatomia, histologia, embryologia, 41(1), 7-11.
109. Leonard, B. C., Cosert, K., Winkler, M., Marangakis, A., Thomasy, S. M., Murphy, C. J., ... & Raghunathan, V. K. (2019). Stromal collagen arrangement correlates with stiffness of the canine cornea. Bioengineering, 7(1), 4.
110. Ribeiro, A. P., Silva, M. L., Rosa, J. P., Souza, S. F., Teixeira, I. A., & Laus, J. L. (2009). Ultrasonographic and echobiometric findings in the eyes of Saanen goats of different ages. Veterinary ophthalmology, 12(5), 313-317.
111. Cafaro, T. A., Ortiz, S. G., Maldonado, C., Espósito, F. A., Croxatto, J. O., Berra, A., ... & Serra, H. M. (2009). The cornea of Guinea pig: structural and functional studies. Veterinary ophthalmology, 12(4), 234-241.
112. Bapodra, P., & Wolfe, B. A. (2014). Baseline assessment of ophthalmic parameters in the greater one-horned rhinoceros (rhinoceros unicornis). Journal of Zoo and Wildlife Medicine, 45(4), 859-865.

113. Laus, J. L., & Aldrovani, M. Parâmetros oftálmicos em cachorro-do-mato (Cerdocyon thous, Linnaeus, 1766).
114. Paszta, W., Klećkowska-Nawrot, J. E., & Goździewska-Harłajczuk, K. (2021). Anatomical and morphometric evaluation of the orbit, eye tunics, eyelids and orbital glands of the captive females of the South African painted dog (Lycaon pictus pictus Temminck, 1820)(Caniformia: Canidae). Plos one, 16(4), e0249368.
115. Balthazar da Silveira, C. P., Lima, T. B., Crivelaro, R. M., de Lacerda, L. C., Pádua, I. R., Renzo, R., ... & Laus, J. L. (2018). Ophthalmic parameters in adult lowland paca (Cuniculus paca) raised in captivity. Veterinary ophthalmology, 21(1), 42-47.
