# Supplementary material for: Glaucoma through Animal’s Eyes: Insights from the Evolution of Intraocular Pressure in Mammals and Birds
Source: Animals (Basel). 2022 Aug 10;12(16):2027. doi: 10.3390/ani12162027 (PMC9404445; doi:10.3390/ani12162027)
Supplement: Supplementary file 1 [file animals-12-02027-s001.zip › Table S1.pdf]

Supplementary Table S1

Comparison of phylogenetic signal of each model in mammals and birds using three modes of evolution (Brownian, Pagel's lambda, Early-burst, and Ornstein-Uhlenbeck) and a non-PGLS model. The model with lowest AIC value is in bold.

**Mammal models**	**Non-PGLS**	**PGLS**		
		**O-U**	**Brownian**	**Pagle's Lambda**
$\log_{10}$(Average body mass)	415.24	**400.04**	425.02	406.83
Type of tonometer	305.64	**303.05**	322.18	307.03
Sedation	337.74	**331.43**	348.83	335.38
Habitat + $\log_{10}$(Average body mass)	402.38	**397.48**	425.58	404.19
Diet + $\log_{10}$(Average body mass)	401.15	**400.04**	427.92	406.74
Average blood pressure (systolic) + $\log_{10}$(Average body mass)	202.96	197.62	218.10	**197.27**
$\log_{10}$(Maximum Longevity)+ $\log_{10}$Average body mass)	354.62	**345.43**	373.37	350.04
Maximum diving depth (m) + $\log_{10}$(Average body mass)	63.90	**62.91**	73.20	66.56

**Bird models**	**Non-PGLS**	**PGLS**		
		**O-U**	**Brownian**	**Pagle's Lambda**
Type of tonometer	216.9406	216.4362	229.8666	**211.3738**
$\log_{10}$(Average body mass)+ Habitat + Diet	**254.2264**	268.6067	319.1307	259.0072
$\log_{10}$(Maximum Longevity)	248.736	242.0277	255.7861	**236.8603**
Maximum diving depth	28.19451	**28.95703**	30.97232	29.05488
